# Therapeutic effects of CO_2_ laser therapy for congenital sebaceous nevus

**DOI:** 10.1093/jscr/rjad467

**Published:** 2023-08-17

**Authors:** Rami Dartaha, Afnan W M Jobran

**Affiliations:** Division of Surgery, Plastic Surgery, Private Clinics, Rambam Health Care Campus, Palestine; Faculty of Medicine, Alquds University, Jerusalem, Palestine

**Keywords:** CO_2_ laser, therapy, congenital sebaceous nevus, case report

## Abstract

Nevus sebaceous (NS) presents as alopecia and yellowish discoloration during infantile stage. In adult stage, lesions become verrucous. Importantly, various appendageal tumors such as trichoblastoma, syringocystadenoma papilliferum and basal cell carcinoma develop during this stage. Hence it is very important to follow the course of NS for early detection of neoplasms. We are presenting a case of a 10-year-old patient with a dome-shaped, dark-pigmented nodule on the left side of neck nape, which later diagnosed as NS and removed with a carbon dioxide laser.

## INTRODUCTION

Jadassohn first identified nevus sebaceous (NS) as a concentric hamartomatous lesion made up of sebaceous glands in 1895 [1]. Pinkus coined the term ‘organoid nevus’ to describe the condition because it affects sweat glands, hair follicles and sebaceous glands in addition to sebaceous glands [2]. During the infancy stage, NS manifests as a well-defined area of alopecia with a smooth surface and a yellowish tint [3]. Adolescence is the time when the organoid nevus enters its second stage of development. During this stage, the lesion’s thickness increases and it may eventually develop a smooth surface with nodularities [4].

Lesions become more verrucous and lobulated in the adult or third stage. It is important to note that during this stage, a variety of appendageal tumors, both benign and malignant, like trichoblastoma, syringocystadenoma papilliferum and basal cell carcinoma, form [5, 6]. As a patient gets older, tumors progress more quickly and occur more frequently [7]. Malignancies are diagnosed in 1% of NS cases, although benign tumors are detected in roughly 13.6% of those cases [5]. In order to diagnose neoplasms early, it is crucial and even required to follow the course of NS.

## CASE PRESENTATION

**Figure 1:**
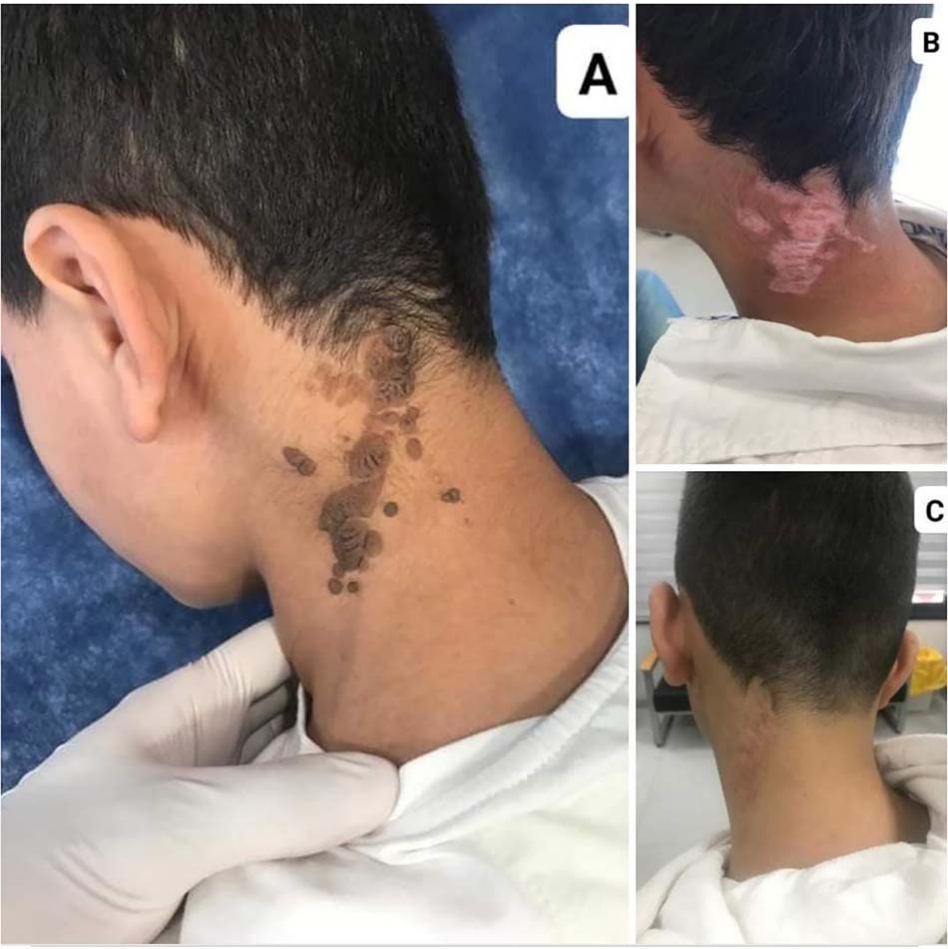
(**A**) A dome-shaped, dark-pigmented nodule on the left side of neck nape NS. (**B**) NS 3 months after treatment. (**C**) NS 1 year after treatment.

A 10-year-old healthy male presented with a dome-shaped, dark-pigmented nodule on the left side of neck nape (Fig. 1A). The pigmented nodule began to appear 1 year ago and enlarged gradually. The patient had no personal or family history of cutaneous or internal malignancies. Physical examination of the revealed a 4 × 10 cm dark, hairless plaque. Systemic examination was found to be normal.

A diagnosis of NS of the neck nape was made following the history and physical examination of the area.

We chose carbon dioxide (CO_2_) laser therapy because of the extensiveness of skin lesions and the effectiveness and accessibility of the method. One-year follow-up shows no recurrence (Fig. 1C).

## DISSCUSSION

Three evolutionary stages that overlap one other make up NS’s natural history and entail differentiation [2]. Yellow-lobed structures, which are vivid yellow patches not connected to hair follicles, are a distinctive feature of the infancy stage of NS [8]. Infantile NS are challenging to distinguish from other congenital lesions on the scalp. Clinically, aplasia cutis, particularly the membrane variety, resembles the infantile stage of NS. Tosti and Piraccini [9] published a study outlining the trichoscopic patterns of aplasia cutis. Neri *et al.* [8] described a situation in which an infant’s early sebaceous nevus and aplasia cutis congenita could be distinguished using in trichoscopy. However, in this investigation, the scientists were unable to see the infantile stage of NS.

Trichoscopy in the early childhood stage revealed yellowish globules with a ‘cobblestone pattern’. In the histology, yellowish globules represent dermal conglomerations of many, hyperplastic sebaceous glands. Additionally, sebaceous hyperplasia and sebaceous adenoma have yellowish globules [10]. The vessels in all of these tumors that are pushed toward the periphery by hyperplastic glands are referred to as ‘crown vessels’. However, the ‘cobblestone pattern’ that was explicitly noted in this study was not described by the authors in their paper. However, this study could not identify any crown vessels.

In the literature, there are no reports of trichoscopy in the adult stage of NS. Trichoscopy of the NS in the adult stage revealed cracks and ridges that were placed in a ‘cerebriform pattern’. In this stage, the hue of the globules changes to brown. According to the histology, this pattern is related to epithelial hyperplasia and papillomatosis. Despite the presence of this pattern, seborrheic keratosis is characterized by comedo-like holes, moth-eating borders, milia-like cysts and telangiectasia [11]. In NS, these are glaringly absent.

NS can be permanently removed with full-thickness excision, just like other epidermal nevi, in patients who express esthetic and emotional pain [12, 13]. The excision of lesions for preventive reasons is still hotly contested [12]. The article by Wali, Felton and McPherson, published in 2018, aims to highlight and assess this discussion. It discusses research done using a questionnaire sent to dermatologists and plastic surgeons in the United Kingdom with the goal of determining the best current intervention for the management of NS [14]. In contrast, only a third of dermatologists agreed that prophylactic excision was the best course of action. This is because the outcomes between the two groups of specialists were different [14]. Moreover, compared with dermatologists who preferred to wait until adulthood, plastic surgeons more frequently suggested that the excision be carried out in childhood [14]. Because of the patient’s significant skin lesions, as well as the treatment’s efficiency and accessibility, we decided to use CO_2_ laser therapy. Patient compliance is better as it is done under local anesthesia in order to avoid any complication of general anesthesia. Post-CO_2_ laser treatment by laser there is no risk for wound dehiscence or open stiches, less infection or swelling-collection.

It preferred to use (co2laser) specially when the lesion is big to avoid big scars or skin tension-deformity-hypertrophic scars. However, because of the possibility of local relapses and malignant transformation inside residual lesions, choosing this therapeutic strategy necessitates further follow-up.

## CONCLUSION

A number of the only procedure offering a complete excision and oncologic safety for NS is surgical removal. Other recommended treatment methods for NS include photodynamic therapy, dermabrasion, laser therapy and cryotherapy. An alternative to early surgical removal of skin lesions is still clinical surveillance. Because of the patient’s significant skin lesions, as well as the treatment’s efficiency and accessibility, we decided to use CO_2_ laser therapy. However, because of the possibility of local relapses and malignant transformation inside residual lesions, choosing this therapeutic strategy necessitates further follow-up.
